# Genetic polymorphisms of *cytochrome P4501A1* and oesophageal squamous-cell carcinoma in Taiwan

**DOI:** 10.1038/sj.bjc.6600499

**Published:** 2002-08-27

**Authors:** M-T Wu, J-M Lee, D-C Wu, C-K Ho, Y-T Wang, Y-C Lee, H-K Hsu, E-L Kao

**Affiliations:** Graduate Institute of Occupational Safety and Health, Kaohsiung Medical University, Taiwan; Department of Occupational Medicine, Kaohsiung Medical University Hospital, Kaohsiung, Taiwan; Department of Surgery, National Taiwan University Hospital, Taipei, Taiwan; Department of Gastroenterology, Kaohsiung Medical University Hospital, Kaohsiung, Taiwan; Department of Chest Surgery, Kaohsiung Veterans General Hospital, Kaohsiung, Taiwan; Department of Chest Surgery, Kaohsiung Medical University Hospital, Kaohsiung, Taiwan

**Keywords:** oesophageal cancer, squamous-cell-carcinoma, *cytochrome p4501A1*, genetic polymorphism

## Abstract

Several *in vitro* studies have demonstrated that genetic polymorphisms result in functionally significant changes in *cytochrome p4501A1* (either *CYP1A1 Msp*I or *exon 7*) but the few epidemiologic studies of these polymorphisms in oesophageal squamous-cell carcinoma have been inconclusive. These inconclusive results motivated us to further examine the relationship between *CYP1A1 Msp*I and *exon 7* polymorphisms and risk of oesophageal cancer. In total, 146 cases of oesophageal squamous-cell-carcinoma and 324 control cases (a total of 470 cases) were genotyped from records at three Taiwan hospitals. No significant association was noted for the *CYP1A1 Msp*I polymorphism variable between carcinoma and control cases. In contrast, the frequency of *Ile/Ile*, *Ile/Val*, and *Val/Val* in *exon 7* was 68 (46.6%), 62 (42.5%), and 16 (11.0%) in carcinoma cases and 179 (55.3%), 127 (39.2%), and 18 (5.6%) in control cases, respectively. After factoring out other potential contributing factors, patients with *Val/Val* showed a 2.48 (95% CT=1.15–5.34) greater risk of developing oesophageal cancer than those with *Ile/Ile*. A slightly (albeit not significantly) greater risk was identified in subjects with *Ile/Val* (OR=1.34; 95% CI=0.86–2.07). These findings suggest that an *exon 7* polymorphism, not a *Msp*I polymorphism, in *CYP1A1* may be pivotal in the development of oesophageal cancer.

*British Journal of Cancer* (2002) **87**, 529–532. doi:10.1038/sj.bjc.6600499 www.bjcancer.com

© 2002 Cancer Research UK

## 

The *CYP1A1* gene code in the aryl hydrocarbon hydroxylase (AHH) enzyme is closely associated with the metabolism of polycyclic aromatic hydrocarbons (PAHs) carcinogens (Crofts *et al*, 1994). Previous studies have suggested that variant alleles of *CYP1A1* *Msp*I polymorphism are associated with malignancies, particularly lung cancer (Kawarjiri *et al*, 1990; Nakachi *et al*, 1991; Hayashi *et al*, 1992; Xu *et al*, 1996). As reported by Bartsch *et al* (2000), no association was identified between *CYP1A1* *Msp*I and *exon 7* polymorphisms and oesophageal cancer risk in a series of studies done on populations of Caucasians and Japanese (Lucas *et al*, 1996; Hori *et al*, 1997; Morita *et al*, 1997; van Lieshout *et al*, 1999). However, Nimura *et al* (1997) studied 89 oesophageal carcinoma patients and 137 cancer-free control patients in an ethnically Chinese population and reported that heavy smokers with *Val/Val* genotypes of *CYP1A1 exon 7* had a three-fold risk of developing oesophageal cancer as compared to those with *Ile/Ile* genotypes. A subsequent study by Roth *et al* (2000) did not find any significant effect of *CYP1A1 exon 7* polymorphisms in 56 individuals with mild or moderate squamous dysplasia and 56 control individuals (a relatively small sample size) from Linxian, a region of high oesophageal cancer risk in China. Recently, Wang *et al* (2002) genotyped 127 oesophageal cancer cases and 101 controls and found that individuals with the *CYP1A1 Val/Val* genotype had a higher risk of developing oesophageal cancer than those with *Ile/Ile* (OR=2.48, 95%CI=1.12–5.54). In view of the apparently significant influence of ethnicity in previous studies, we have examined further the role of *CYP1A1* *Msp*I and *exon 7* polymorphisms in oesophageal cancer risk in Taiwan.

## MATERIALS AND METHODS

### Selection of cases and controls

Over the four-year period of this study, we recruited 146 patients with pathologically-proven oesophageal squamous-cell-carcinoma undergoing treatment at three hospitals in Taiwan, including the National Taiwan University Hospital, the Kaohsiung Medical University Hospital, and the Kaohsiung Veterans General Hospital. Concurrently, the preventive medicine department at each hospital did their best to also recruit 1–4 malignancy-free subjects per recruited carcinoma patient as healthy controls (*n*=324). Healthy control subjects were selected based on being the same gender as the recruited carcinoma patient and being in the hospital during the same time and of roughly the same age (±3 years). Subjects in this study were interviewed by trained interviewers using a standardized questionnaire to collect demographic and substance use (cigarette, alcohol, and areca) information. This study was approved by National Taiwan University's IRB Hospital. Informed consent was obtained from all subjects.

## *CYP1A1 Msp*I polymorphism

The *Msp*I polymorphism in the *CYP1A1* 3′ flanking region was determined using PCR and RFLP (Wu *et al*, 1998). The DNA sample was amplified with 2 primers: 5′-CAGTGAAGAGGTGTAGCCGC-3′ (upstream) and 5′-TAGGAGTCTTGTCTCATGCC-3′ (downstream) (Perkin Elmer, Taipei, Taiwan). Amplification was performed by initial denaturation at 94°C for 5 min, followed by 30 cycles at 94°C for 1 min, 61°C for 1 min, 72°C for 1 min, and a final extension at 72°C for 7 min.

The 10-μl amplification result was digested using 3 units *Msp*I (New England Biolabs, Beverly, MA, USA). When an *Msp*I restriction site was present, the fragment of 340 bp was digested into two lengths of 140- and 200-bp. Homozygous wild-type individuals lacked the 140- and 200-bp fragment, and heterozygous individuals had three bands; homozygous-rare allele individuals lacked the large parent band while showing the smaller bands.

### *CYP1A1 Ile/Val* polymorphism

The *Bsr*DI polymorphism in *CYP1A1 exon 7* was determined using PCR and RFLP, according to the method used by Cascorbi *et al* (1996), albeit with slight modifications. The DNA sample was amplified with two primers: 5′-CTGTCTCCCTCTGGTTACAGGAAGC-3′ (upstream) and 5′-TTCCACCCGTTGCAGCAGGATAGCC-3′ (downstream) (Perkin Elmer, Taipei, Taiwan). Amplification was performed by initial denaturation at 95°C for 5 min, followed by 35 cycles at 95°C for 30 s, 73.5°C for 30 s, 72°C for 30 s, and a final extension at 72°C for 7 min. The final product was digested by *Bsr*DI. When a *Bsr*DI restriction site was present, the fragment of 204-bp was digested into two lengths of 65- and 139-bp. Individuals with *Ile/Ile* had the 65- and 139-bp fragment, heterozygous individuals had three bands; and individuals with *Val/Val* retained the larger parent bands (204-bp).

### Laboratory QA/QC

We included one positive control and one negative control samples in each genotyping set (∼10 samples). The positive control sample was included to ensure complete digestion of the PCR product by restriction enzymes. The negative control was placed with the same reagents as those used with actual samples, with the exception of DNA templates.

### Statistical analysis

The gene frequency and Hardy-Weinberg equilibrium tests were conducted, with results entered into multiple logistic regression models to determine, after adjustments had been made for other factors of influence, the level of association between *CYP1A1 Msp*I and *exon 7* polymorphisms and oesophageal cancer risk. Factors of influence (covariates) in the models included the variables of age (>65 years and ⩽65 years), gender, education level (⩾college, high school, and ⩽elementary school), and race (Fukienese, Mainlander, and ‘other’). The data were analysed using the SAS statistical package and all *P*-values were two-sided.

## RESULTS

The mean age for carcinoma and control cases was 60.6 and 61.2 years, with an associated age range of 37 to 81 and 34 to 84 years, respectively. The habitual use of cigarettes (*P*=0.03), alcohol (*P*=0.03), and areca (*P*<0.01) provided the most significant predictors of oesophageal cancer risk (Table 1

**Table 1:**
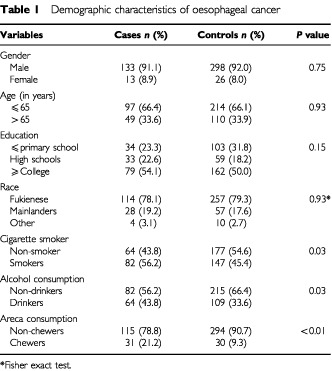
Demographic characteristics of oesophageal cancer

).

The frequency of *Msp*I polymorphism in wild-type, heterozygous variant, and homozygous variant control cases was 136 (42.0%), 146 (45.1%), and 42 (13.0%), respectively. The gene frequency of the variant allele was 35.5%. The frequency of *Ile/Ile*, *Ile/Val*, and *Val/Val* in *exon 7* among controls was 179 (55.3%), 127 (39.2%), and 18 (5.6%), respectively. The gene frequency of the variant allele (*Val*) was 25.2%. The distribution of the different genotypes in both *Msp*I and *exon 7* polymorphisms among the 324 control cases closely conformed to Hardy-Weinberg expected frequencies (χ^2^=0.04; d.f.=2; *P*=0.98 and χ^2^=0.28; d.f.=2; *P*=0.87, respectively).

The frequency of *Ile/Ile*, *Ile/Val*, and *Val/Val* in *exon 7* was 68 (46.6%), 62 (42.5%), and 16 (11.0%) in carcinoma cases and 179 (55.3%), 127 (39.2%), and 18 (5.6%) in control cases, respectively. As lists of those genotypes with elevated oesophageal cancer risk were similar both after adjusting only for substance use and after adjusting for substance use along with age, gender, education, and race, we present here the results for the latter category. Compared to those with *Ile/Ile*, subjects carrying *Val/Val* had a 2.34-fold risk (95% CI=1.13–4.85) of developing oesophageal cancer. After adjusting for other potential confounding factors, the result remained similar (OR=2.48; 95% CI=1.15–5.34) (Table 2

**Table 2:**
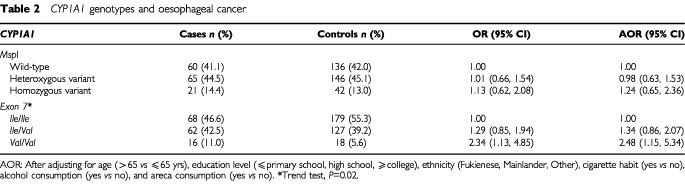
*CYP1A1* genotypes and oesophageal cancer

). The results show even a slightly greater risk after further adjusting for *Msp*I polymorphism (OR=2.71; 95% CI=1.75–6.38). A slight, but not significant, risk elevation was found in subjects with *Ile/Val* both before and after adjusting for confounding factors (OR=1.29 and 1.34; 95% CI=0.85–1.94 and 0.86–2.07, respectively). In contrast, we found no significant polymorphic effect of *CYP1A1 Msp*I on oesophageal cancer (Table 2).

## DISCUSSION

The results of this study found that those cases with the *CYP1A1 Val/Val* genotype had a 2.34 times higher risk (95% CI=1.13–4.85) of developing oesophageal cancer than those with the *Ile/Ile* genotype. In contrast, the *CYP1A1 Msp*I genetic polymorphism was not found to be associated with elevated oesophageal cancer risk. Several studies on ethnic Japanese populations reported no association between *CYP1A1* *Msp*I and *exon 7* polymorphisms and risk of oesophageal cancer (Hori *et al*, 1997; Morita *et al*, 1997; van Lieshout *et al*, 1999). However, their small case population (less than 100 oesophageal cancer cases were investigated) may not be sufficient to make a scientific determination regarding genotype significance. Although Roth *et al* (2000) found no significant effect of *CYP1A1 exon 7* polymorphisms on either the 56 individuals with mild or moderate squamous dysplasia or the 56 control cases from a high oesophageal cancer risk region with an ethnic Chinese population, Nimura *et al* (1997) found subjects in China with heavy smoking habits had a three times higher frequency of the *CYP1A1 Val/Val* variant. Recently, Wang *et al* (2002) also found that individuals with the *CYP1A1* *Val/Val* genotype had a higher risk of developing oesophageal cancer than those with the *Ile/Ile* (OR=2.48, 95% CI=1.12–5.54). Our findings approximate the Wang *et al* (2002) findings.

Human cancers, e.g., gastrointestinal cancers, were suggested to be the result of the activation of procarcinogens into carcinogens (Nakajima *et al*, 1996). Murray *et al* (1994) and Nakajima *et al* (1996) examined the expression of cytochrome P450s in human esophagi with squamous-cell carcinomas. Both studies found that the amount of cytochrome P450 1A1 expression in tumorous tissue to be significantly higher than that in non-tumorous tissues. These two findings suggest that the inducible *CYP1A1* gene might contribute to oesophageal cancer development. In two *in vitro* studies, Kiyohara *et al* (1996) examined the relationship between AHH inducibility (3-methylcholanthrene (MC)-induced AHH activity/non-induced AHH activity) and the frequency of *CYP1A1* *Msp*I and *exon 7* polymorphisms in peripheral lymphocytes in 84 healthy Japanese male subjects *in vitro*. They found the age-adjusted AHH inducibility (mean±standard error) of the wild-type, heterozygous, and homozygous variants of the *CYP1A1 Msp*I gene to be 4.89±0.36, 4.82±0.29, and 13.61±1.44, respectively. Furthermore, the homozygous variants had significantly higher AHH inducibility than the combined wild-type and heterozygous variants. However, no association was found between AHH inducibility and *CYP1A1 exon 7* polymorphisms. In contrast, Crofts *et al* (1994) measured gene expression levels and AHH enzymatic activity levels of *Msp*I and *exon 7* polymorphisms in mitogen-stimulated lymphocytes in 51 healthy subjects. They reported that subjects with the *exon 7* polymorphism (variant alleles) (*n*=12) had a relative level of *CYP1A1* mRNA inducibility (induced/basal) of 9.0±1.7, versus 5.9±0.6 in people with the wild-type alleles (*n*=39). However, variant genotypes at the *Msp*I site had no effect on *CYP1A1* gene induction. Our results in this epidemiologic study align more closely with those obtained by Crofts *et al* (1994).

Our previous study found the prevalence (number) of homozygous wild-types, heterozygous variants, and homozygous variants in *CYP1A1 Msp*I polymorphisms to be 42.5% (34), 42.5% (34), and 15.0% (12), respectively, among 80 coke-oven workers (Wu *et al*, 1998). This distribution parallels the control population recruited for our present study, suggesting no potential selection bias was present.

In summary, this study found *CYP1A1 exon 7* (but not *Msp*I) polymorphism to be a factor in elevated oesophageal cancer risk. This association suggests that carcinogen biotransformation may be a contributing factor to the etiology of oesophageal squamous-cell-carcinoma among the population in Taiwan.

## References

[bib1] BartschHNairURischARojasMWikmanHAlexandrovK2000 Genetic polymorphism of CYP genes, alone or in combination, as a risk modifier of tobacco-related cancers Cancer Epidemiol Biomarkers Prev 9 328 10667460

[bib2] CascorbiIBrockmollerJRootsI1996 A C4887A polymorphism in exon 7 of human CYP1A1: population frequency, mutation linkages, and impact on lung cancer susceptibility Cancer Res 56 49654969 8895751

[bib3] CroftsFTaioliETrachmanJCosmaGNCurrieDTonioloPGarteSJ1994 Functional significance of different human CYP1A1 genotypes Carcinogenesis 15 29612963 8001264 10.1093/carcin/15.12.2961

[bib4] HayashiSWatanabeJKawarjiriK1992 High susceptibility to lung cancer analyzed in terms of combined genotype P4501A1 and Mu-class glutathione-S-transferase genes Jpn J Cancer Res 83 866870 1399823 10.1111/j.1349-7006.1992.tb01992.x PMC5918950

[bib5] HoriHKawanoTEndoMYuasaY1997 Genetic polymorphisms of tobacco- and alcohol-related metabolizing enzymes and human oesophageal squamous cell carcinoma susceptibility J Clin Gastroenterol 25 568575 9451664 10.1097/00004836-199712000-00003

[bib6] KawarjiriKNakachiKImaiKYoshiiAShinodaNWatanabeJ1990 Identification of genetically high risk individuals to lung cancer by DNA polymorphisms of the cytochrome P4501A1 gene FEBS Lett 263 131133 1691986 10.1016/0014-5793(90)80721-t

[bib7] KiyoharaCHirohataTInutsukaS1996 The relationship between aryl hydrocarbon hydroxylase and polymorphisms of the CYP1A1 gene Jpn J Cancer Res 87 1824 8609043 10.1111/j.1349-7006.1996.tb00194.x PMC5920980

[bib8] LucasDMenezCFlochFGourlaouenYSparfelOJoannetIBodenezPJezequelJGouerouHBerthouFBardouLGMenezJF1996 Cytochromes P4502E1 and P4501A1 genotypes and susceptibility to cirrhosis or upper aerodigestive tract cancer in alcoholic caucasians Alcohol Clin Exp Res 20 10331037 8892524 10.1111/j.1530-0277.1996.tb01943.x

[bib9] MoritaSYanoMShiozakiHTsujinakaTEbisuiCMorimotoTKishibutiMFujitaJOgawaATaniguchiMInoueMTamuraSYamazakiKKikkawaNMizunoyaSMondenM1997 CYP1A1, CYP2E1 and GSTM1 polymorphisms are not associated with susceptibility to squamous-cell carcinoma of the esophagus Int J Cancer 71 192195 9139841 10.1002/(sici)1097-0215(19970410)71:2<192::aid-ijc11>3.0.co;2-k

[bib10] MurrayGIShawDWeaverRJMcKayJAEwenSWMelvinWTBurkeMD1994 Cytochrome P450 expression in oesophageal cancer Gut 35 599603 8200549 10.1136/gut.35.5.599 PMC1374739

[bib11] NakachiKImaiKHayashiSWatanabeJKawarjiriK1991 Genetic susceptibility to squamous cell carcinoma of the lung in relation to cigarette smoking dose Cancer Res 51 51775180 1655248

[bib12] NakajimaTWangRSNimuraYPinYMHeMVainioHMurayamaNAoyamaTIidaF1996 Expression of cytochrome P450s and glutathione S-transferases in human esophagus with squamous-cell carcinomas Carcinogenesis 17 14771481 8706252 10.1093/carcin/17.7.1477

[bib13] NimuraYYokoyamaSFujimoriMAokiTAdachiWNasuTHeMPingYMIidaF1997 Genotyping of the CYP1A1 and GSTM1 genes in oesophageal carcinoma patients with special reference to smoking Cancer 80 852857 9307183 10.1002/(sici)1097-0142(19970901)80:5<852::aid-cncr4>3.0.co;2-n

[bib14] RothMJDawseySMWangGTangreaJAZhouBRatnasingheDWoodsonKGOliveroOAPoirierMCFryeBLTaylorPRWestonA2000 Association between GSTM1*0 and squamous dysplasia of the esophagus in the high risk region of Linxian, China Cancer Lett 156 7381 10840162 10.1016/s0304-3835(00)00442-0

[bib15] van LieshoutEMRoelofsHMDekkerSMulderCJWobbesTJansenJBPetersWH1999 Polymorphic expression of the glutathione S-transferase P1 gene and its susceptibility to Barrett's esophagus and oesophageal carcinoma Cancer Res 59 586589 9973204

[bib16] WangAHSunCSLiLSHuangJYChenQS2002 Relationship of tobacco smoking CYP1A1 GSTM1 gene polymorphism and oesophageal cancer in Xi'an World J Gastroenterol 8 4953 11833070 10.3748/wjg.v8.i1.49 PMC4656624

[bib17] WuMTHuangSLHoCKYehYFChristianiDC1998 Cytochrome P450 1A1 MspI polymorphism and urinary 1-hydroxypyrene concentrations in coke-oven workers Cancer Epidemiol Biomarkers Prev 7 823829 9752993

[bib18] XuXKelseyKTWienckeJKWainJCChristianiDC1996 Cytochrome P450 CYP1A1 MspI polymorphism and lung cancer susceptibility Cancer Epidemiol Biomark Prev 5 687692 8877059

